# Current opinions and recommendations of paediatric healthcare professionals – The importance of tablets: Emerging orally disintegrating versus traditional tablets

**DOI:** 10.1371/journal.pone.0193292

**Published:** 2018-02-28

**Authors:** Hamad Alyami, Jasdip Koner, Chi Huynh, David Terry, Afzal R. Mohammed

**Affiliations:** 1 Aston Pharmacy School, Aston University, Birmingham, United Kingdom; 2 Academic Practice Unit, Birmingham Children’s Hospital, Birmingham, United Kingdom; University College London, UNITED KINGDOM

## Abstract

The appropriate prescribing of paediatric dosage forms is paramount in providing the desired therapeutic effect alongside successful medication adherence with the paediatric population. Often it is the opinion of the healthcare practitioner that dictates which type of dosage form would be most appropriate for the paediatric patient, with liquids being both the most commonly available and most commonly used. Orally disintegrating tablets (ODTs) are an emerging dosage form which provide many benefits over traditional dosage forms for paediatric patients, such as rapid disintegration within the oral cavity, and the reduction in the risk of choking. However the opinion and professional use of healthcare practitioners regarding ODT’s is not known. This study was designed to assess the opinions of several types of healthcare professionals (n = 41) regarding ODTs, using a survey across two hospital sites. Results reaffirmed the popularity of liquids for prescribing in paediatrics, with 58.0% of participants preferring this dosage form. ODTs emerged as the second most popular dosage form (30.0%), with healthcare practitioners indicating an increasing popularity amongst patients in the hospital setting, belief with 63.0% of practitioners agreeing that many liquid formulations could be substituted with a suitable ODT. The desired properties of an ideal ODT were also identified by healthcare practitioners preferring a small, fast disintegrating tablet (90.2% and 95.1% respectively), with the taste, disintegration time and flavour being the three most important attributes identified (29.5%, 28.7% and 21.7% respectively). This study provided a pragmatic approach in assessing healthcare professional’s opinions on ODTs, highlighting the ideas and thoughts of practitioners who are on the frontline of paediatric prescribing and treatment and gave an indication to their preference for ODT properties.

## Introduction

The European Medicines Agency (EMA) recognised that there was limited data concerning paediatric population acceptance of oral dosage forms in relation to age and developmental status, along with the inadequate availability of licensed medicines appropriate for administration to children [1]. In addition, there is anecdotal evidence that there are increasing concerns amongst healthcare professionals about paediatric patients failing to take their prescribed medication [2]. Many medicines are formulated to enable usage in adults, which may not be suitable for use by children. There may be difficulties in swallowing solid dosage forms (e.g. tablets) and there may be issues concerning the availability of the dose strength, based on current dosage forms available. Many children will require doses smaller than adults, prompting the use of liquids or splitting the dose of solid tablets by cutting them into halves and quarters. If suitable dosage forms are not available then patient compliance with prescribed medication may be reduced with potential adverse clinical consequences [3].

Reasons that may affect a child’s success in swallowing solid dosage forms include developmental stage, depending on their age (0 to 18 years), anxiety, fear, intolerance to unpleasant flavours and not being able to appreciate the risks associated with noncompliance [4].

Treatment failure may result, leading to poor clinical control and unnecessary expense as a result of unused medication waste. In primary care around £300 million worth of medicines are wasted every year of which £150 million is preventable [5]. Formulation work so far in pharmaceutical industries has revealed that liquids seem to be more customary with the paediatric population (infant age between 1 month to 2 years and pre-school age between 2 to 5 years), whereas oral disintegrating tablets (ODT) may be preferred by those who are older (5 to 11 years), and in the adolescent age (12 to 16/18 years), tablets and capsules may be more appropriate and convenient [6]. An ODT is an easy to use dosage form which disintegrates in the mouth upon contact with saliva. ODTs can be taken without the need to swallow the tablet whole and does not require water. Advancements in the area of ODT formulation were aimed equally at escalating the performance of the dosage form by lessening the disintegration time, and by increasing the compliance of patients via masking the unpleasant taste of the API. These successes require stable improvement of formulation variables, together with technologies concerned in the manufacture of dosage forms. The inclusion of super-disintegrants to produce efficient ODTs is not new. Conversely, with the development design of innovative techniques, it has become promising to formulate ODTs with less content of super disintegrants and with improved mouth feel [6]. There is evidence to suggest that ODTs are a potential ideal formulation for children since they avoid concerns children may have regarding swallowing tablets. [7, 8].

A previous study by the authors was conducted in three countries Jordan, Saudi Arabia and the UK found that approximately 58.0% of the participants (children age 6–18 years) preferred taking ODTs compared to conventional tablets, liquids and capsules [9].

Few studies have been carried out aimed at identifying healthcare professional perceptions or opinions regarding the use of different dosage forms for paediatric use. However, previous research conducted in similar fields has explored healthcare professional’s perceptions on HIV treatment adherence in children with an investigation in to unlicensed/off label medicines use [10] and those exploring paediatric nurses knowledge and practice of mixing medicines with foodstuffs [11]. Most research in this field is targeted at reducing prescribing and dispensing errors for children. However, to ensure medication adherence in children is supported, when making a decision on medication formulation choice for a child, clinicians should take into consideration the acceptability of the dosage form to paediatric patients. To the best of our knowledge there are limited published studies regarding the opinions of healthcare providers concerning ODTs preparations. As ODTs are an emerging dosage form which is increasing in popularity, it is important to gather opinions of paediatric healthcare professionals as to their prescribing habits and also preference for ODTs. Their opinion on the desired characteristics is important as this may reflect their prescribing/administration of the dosage form. Furthermore, limited data is available concerning the effect of ODTs properties (i.e. organoleptic, taste (sweet, sour, bitter, salty or umami), texture, flavour, colour, shape, size and disintegration time) on child acceptance. Poor acceptance could lead to a child resisting or rejecting a medication. The palatability of paediatric oral medicines is one of the most important factor with potential to impact adherence to therapeutic regimens. Drug design and manufacture goes beyond the basic principles of producing drugs with the best therapeutic profile. It also involves the manufacture of drugs whose taste is acceptable to the target age group, in this case the paediatric population. Drugs, which are not palatable, have lower acceptability. For this reason, the taste may be masked or the drug flavoured to ‘conceal’ the bad taste. Flavouring involves adding substances that give the drug a characteristic taste and smell, different from that of the actual drug. Taste addiction is a situation in which the patient develops a liking for the taste of the drug and feels an urge to take the drug often. Although taste addiction may resolve the issue of non-adherence, it may result in grave consequences such as abuse of the drug. Care should be taken to avert this. The flavouring is important because it tailors the taste and smell of the drug to the convenience of the target age group. Previous results obtained from research studies on the popular flavours aid in developing drugs with specific flavours and which suit the needs of the target age group [1, 3,9].

To complement and supplement the findings of formulation preference in children [7], and to gather the opinion of healthcare professionals responsible for the care of a child in terms of prescribing, supplying and administering their medication, it was therefore necessary to conduct a study exploring the opinions of healthcare professionals regarding paediatric dosage forms, and in particular ODTs.

The primary aim of the present work is to evaluate healthcare professional’s perceptions of the paediatric dosage forms to support patient choice and ultimately patient adherence to prescribed medication regimens.

The main objective of the focus group and semi-structured interviews (phase 1 and 2 respectively) was to design a validated online survey (phase 3) delivered to pharmacists, nurses and medical practitioners to evaluate their views and perceptions with regards to paediatric dosage forms. This study will provide the opinion of healthcare professionals in what dosage forms they believe are preferred by children and to also identify healthcare professional’s personal opinions concerning the safety and cost effectiveness of formulation types. The secondary aim of this study was to compare the findings of this present study concerning healthcare professionals with the findings from the previous study concerning children in respect to dosage forms [9].

## Methodology and ethical considerations

### Overall methodological design of the study

The study consisted of dual site, cross-sectional, mixed methods study of hospital based paediatric doctors, pharmacists and nurses, using an anonymised electronic survey (Bristol Online Survey software–BOS); informed by a literature search, focus groups and semi-structured interviews.

This study was carried out at two UK city based paediatric hospitals in the West Midlands and North West of England, in this article named as Hospital 1 and Hospital 2.

Informed by a literature search, this study included a three-phase consensus-building process comprising of

- Phase (1) focus groups with pharmacists and nurses (separately);
- Phase (2) semi-structure face-to-face interviews with each of the three main groups of professionals, consent forms were given to focus group phase (1) and semi-structure interviews phase (2) and
- Phase (3) electronic survey of paediatric hospital healthcare professionals.

Qualitative verbatim transcripts from phases 1 and 2 were subjected to framework analysis [12]. Themes from phase 1 underpinned the basis for phase 2 interviewees. Themes from phases 1 and 2 generated issues for inclusion within phase 3.

The final electronic questionnaire (phase 3) was assembled and managed using bespoke software (Bristol Online Survey ™). Results were transferred to SPSS version 22 and NVivo version 10 software for analysis to facilitate descriptive statistical analysis and framework analysis respectively.

**Fig 1:**
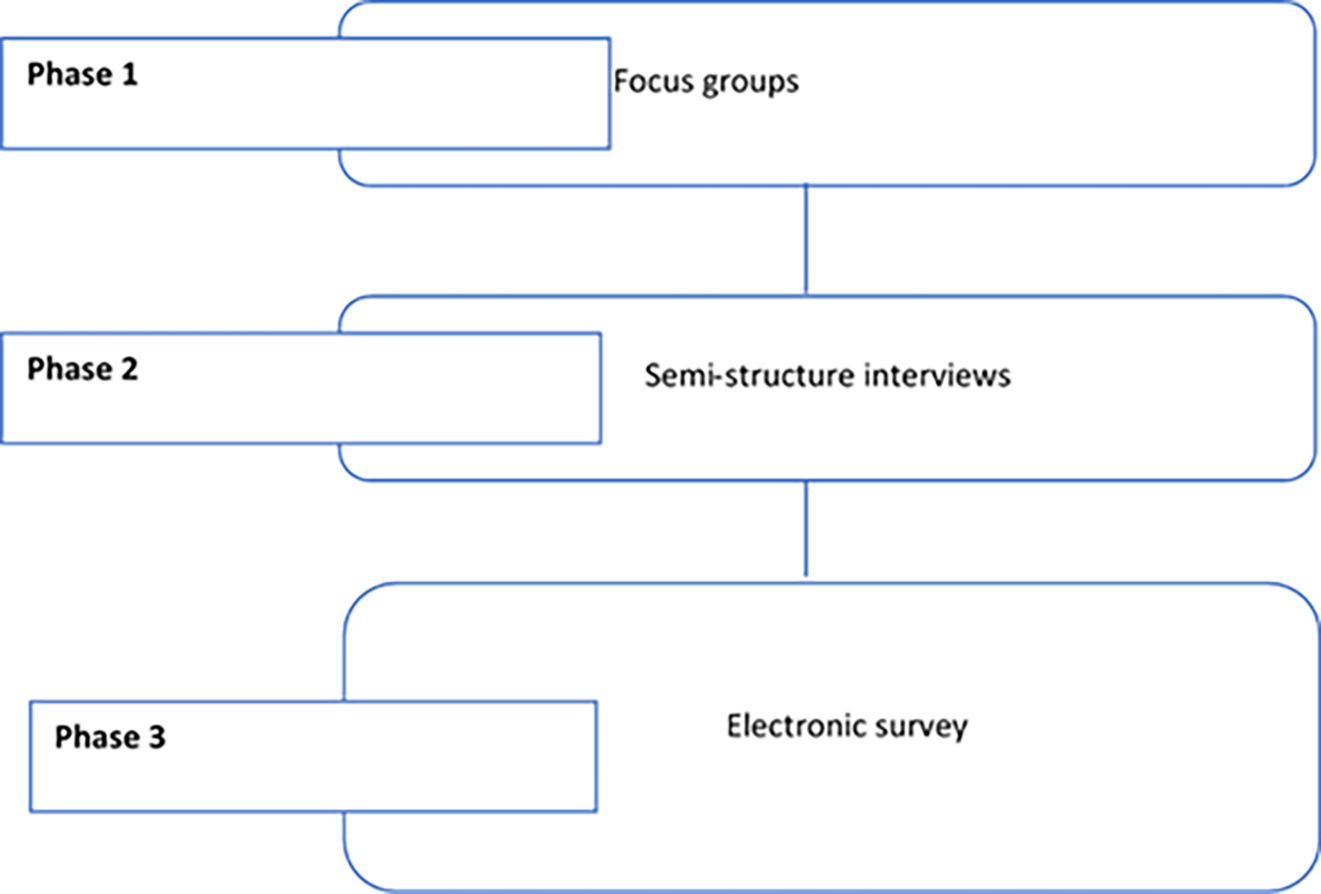
Flow chart of research methodology.

Fig 1 presents a flow diagram of the three phases that were carried out. The questionnaire was comprised of both closed and open questions to identify participants’ perceptions and opinions about dosage forms for children.

### Recruitment and consent

#### Phase 1 –Focus group

Two focus groups were undertaken within this study. One focus group consisted of 4 nurses and one of 7 pharmacists. These focus groups were designed to support the development of the healthcare professionals’ questionnaire. All focus groups were conducted at the primary study site (Hospital 1). Email invitations to attend the focus groups were arranged via the research leads of the two professions at Hospital 1. All potential participants received a Participant Information Sheet and those recruited to the study completed and signed the relevant consent form. Private rooms were pre-booked within Hospital 1 in locations accessible for staff, to create a suitable and convenient environment for discussion. The focus groups were facilitated by HA (Hamad Alyami) and assisted by CH (Chi Huynh) (research pharmacist/lecturer in clinical pharmacy). The groups were digitally audio-recorded. A question guide was developed including questions to be discussed by the facilitator to ensure coverage of pre-determined themes identified by the project support team.

#### Phase 2 –Semi-structured interviews

Participants from the three professional groups were recruited to undertake semi-structured interviews. They were recruited by recommendation from the professional research leads or via their involvement in the Academic Practice Unit at Hospital 1. A minimum of three participants from each of the three professional groups were recruited to the study. All potential participants received a Participant Information Sheet and those recruited to the study completed and signed the relevant consent form.

#### Analysis of the focus groups and semi-structured interviews data

The data collected from Phase 1 and Phase 2 were analysed using framework analysis by [13, 14] with the following stages, 1) Data entry and processing; 2) Familiarisation of the focus group and interview data collection and identifying an initial framework based on the question guide 5); 3) Coding–all focus groups and interviews were coded and indexed using NVIVO 10; 4) Generation of themes (charting) results were charted according to the themes; 5) Finalised framework (mapping and interpretation).

HA transcribed each focus group and semi-structured interview as soon as was possible after facilitating the group and interview. Verbatim transcripts were produced in Microsoft Office Word 2013 from the digital audio-recordings. Participants were anonymously assigned a coded identifier within the text (e.g. speaker 1, speaker 2 etc.) to ensure the extent to which views were shared could not be identified. The verbatim text was copied in to a qualitative data analysis and coding software (QSR NVivo 10) and framework content analysis was developed to analyse the verbatim files. Qualitative research software (NVivo 10) was used to arrange information and combine analysis with linking [15]. A framework analysis approach was used to qualitatively analyse the data [16]. The data was coded using coding framework based on the question guide for the focus group, interviews and objectives of this study (see Table 1). The initial 10 minutes (20%) of the transcript recording, was coded independently by two investigators, HA and CH, using the initial framework with NVivo Version 10 to assist with indexing the codes. The similarity and differences between the two coders was discussed. The differences between the two coders were resolved via discussion of differences until a consensus was reached. A resulting final framework developed. The coding framework was used to code the rest of the transcript. The interpretation of the results from the coding of the focus group and semi-structured interviews were conducted by HA and was based on the objectives of the focus group and interviews.

**Table 1:** Compiled sample of framework coding for focus groups and semi-structured interviews.

Main code	Sub codes (Nvivo nodes)
1. Most common paediatric dosage form	1.1 Liquids
	1.2 Tablets
	1.3 Suspensions
	1.4 ODTs
2. Your preferences for paediatric oral dosage forms	2.1 Tablets
	2.2 Solutions
	2.3 Capsules
	2.4 ODT
	2.5 Mini-tablets
	2.6 Depends on age, patient
3. ODTs as an alternative dosage forms	3.1 Yes, for young children
	3.2 Yes, it is good alternative
	3.3 Children and parents need education regarding ODTs
	3.4 Sometimes not always
	3.5 Should taste good (sweet)
	3.6 Not suitable for less than 6 yrs
4. Enhancement of compliance and adherence regarding taking ODTs	4.1 Absolutely, if it tastes good
	4.2 I think so
	4.3 Taste is going to be important
	4.4 Depends on age/patient
	4.5 Lot of lansoprazole ODTs, it helps
5. ODTs cost effectiveness	5.1 Definitely much cheaper than liquids
	5.2 Much more expensive
	5.3 No idea
6. Any feedback from the patients or their parent regarding any issues of ODTs	6.1 Taste not good, talk about taste
	6.2 Too big in size
	6.3 East of being able to swallow
	6.4 Not really
7. Characteristics of ODTs	7.1 Taste
	7.2 Flavour
	7.3 Size
	7.4 Colour

#### Phase 3 –Electronic survey

An electronic survey tool was chosen as the instrument for this study. Healthcare professionals working at the time of the study for Hospital 1 and Hospital 2 were invited to complete an online survey. Managing of this survey by the use of purpose designed electronic survey software was therefore considered to be both deliverable and efficient due to suitability of having automated data collection, which saves researcher time, effort and offers cost savings advantages [17]. The questionnaire development was supported by the HCPs focus groups and the semi-structured interviews.

The themes and concerns identified in the phase 1 and 2 were considered for inclusion in the survey, including for example factors influencing choice of formulations for paediatric patients. A draft survey was created on 11th August 2016 using Bristol Online Survey software (BOS).

Email addresses of HCPs were obtained from the site leads at Hospital 1 and Hospital 2 (CH and chief pharmacist) respectively and they managed this process by reference to staffing lists and responses. The invitation to participate was sent from Bristol online survey to the NHS Trust email addresses of the study cohort with a link to the survey. The purpose of the survey was explained in the beginning of the survey and informed consent was presumed when the participant decided to complete the survey. First emails were sent out on with reminder emails sent to all respondents 3 and 6 weeks after the initial email was sent. Each site was required to return completed surveys from a minimum of five professionals in each group.

Participants were advised that all data were held confidentiality and anonymity was assured.

Responses were exported from Bristol survey into MS Excel 2013 and IBM SPSS version 22 for analysis and production of descriptive statistics.

### Inclusion and exclusion criteria

Inclusion criteria:

- Healthcare professionals (HCPs) (doctor, nurse, and pharmacist) at Hospital 1.
- Healthcare professionals (doctor, nurse, and pharmacist) at Hospital 2.

Exclusion criteria:

- General public

### Ethical considerations

The study design was approved by both the Aston University School of Life and Health Sciences and the Research & Development Departments at both Hospital 1 and 2. All participants in the study gave informed consent.

Data was only accessible by the study team. All responses were fully anonymised prior to analysis and all reports accommodated confidentiality requirements. Audio files from the focus groups/semi-structured interviews were held on-site at Hospital 1, within the secure area of the Academic Practice Unit. Once transcripts were approved, original recordings were destroyed. Additionally, paper records (from the semi-structured interviews) were also kept within the secure area of the Academic Practice Unit at Hospital 1, and were destroyed upon transcription of the interviews.

## Results and discussion

The presentation of the findings is divided into three phases. The first phase reports the focus groups of healthcare professionals; the second phase addresses the semi-structured interviews and the third phase (main phase) presents the online survey for healthcare professionals concerning paediatric dosage forms. The healthcare professionals that were selected to participate in this study were medical practitioners, pharmacists and nurses. The rational for selection of HCPs was to show the relationship leading from the prescribing (doctor) through to dispensing medicine (pharmacist) and lastly administration (nurse).

### Phase 1- Focus group

The focus groups were used to scope the research and inform design of the online survey with healthcare professionals (phase 3) and aimed at seeking opinions of participants with a different range of backgrounds. One limitation of this method was that participants had to obligate their time to take part. Although it was planned to conduct a focus group for doctors, it was not feasible due to the clinical demands of the service. However, the information gathered from the Hospital 1 focus groups provided an understanding in to the opinions of pharmacists and nurses concerning paediatric dosage forms, whilst the sample size for doctors was proposed to be increased within the semi-structured interviews (phase 2). Table 2 shows the number of participants, dates conducted and location of the focus groups. The pharmacist and nurse groups were conducted at lunchtime, it was intended that each session would last between 30 and 50 minutes. The exact timings of digital audio-recordings are shown in (Table 2) below and discussion flowed well between the group members.

**Table 2:** Details of the two focus groups for healthcare professionals.

Focus group	Date conducted	Number of participants	Location	Time duration of group (minutes)
**Pharmacists**	7^th^ July 2016	7	Hospital 1	43
**Nurses**	14^th^ July 2016	4	Ward 10 Hospital 1	32

It was mentioned by a pharmacist that the most preferred dosage forms for paediatric population age (6–18 years) depends upon the patients and what they would prefer, for example if they were unable to swallow tablets, liquids are the obvious default, but if the liquid was unavailable an alternative would be suitable for example an ODT or crushable.

In addition it was reported that texture and taste of ODTs was important for children so the ideal taste would be sweet with either citrus or strawberry flavours.

Across the focus groups, the large sizes of tablets were related to swallowing difficulties in paediatric patients especially those less than 6 years of age.

Several studies exploring children suffering from HIV support these findings and stated the negative attitudes of children regarding the size of antiretroviral tablets [4, 18, 19].

### Phase 2- Semi-structured interviews

A total of 12 healthcare professionals were interviewed at Hospital 1 during the study period, the HCPs recruited for this phase of the study were predominantly medical staff (2 consultants and 4 junior doctors) (Table 3). All participants answered the questions regarding the paediatric dosage forms followed by properties of ODTs. Phase 2 findings suggested that the main issues with the properties of ODT formulations are those associated with taste, size and disintegration time. However, colour and shape of ODTs were highlighted the least important by 85% of respondents.

**Table 3:** Details of the semi- structured interviews for healthcare professionals.

Semi-structured interviews	Date conducted	Number of participants	Location
**Pharmacists**	18^th^ -25^th^ July 2016	3	Hospital 1
**Nurses**	18^th^ -25^th^ July 2016	3	Hospital 1
**Doctors**	18^th^ -25^th^ July 2016	6	Hospital 1

Additional informal recommendations from healthcare professionals within phase 2 on how to improve ODTs formulations were reported in (Table 4).

**Table 4:** Recommendations and improvements to ODTs formulations as reported by focus groups and semi-structured interviews healthcare professionals.

HCPs recommendations to ODTs formulations	Reports of healthcare professionals
Enhancing the taste of ODT formulations using sweet to neutral sweeteners	Pharmacist FG, Doctor 1, 3, 4 and 5 SI, Pharmacists SI, Nurse 1 and 3 SI
Using strawberry (most preferred), orange or banana flavours.	Nurse FG, Pharmacist 1,2 and 3 SI
Using small size of ODTs	All participants for FG and SI
Improving disintegration time (Dissolving very quickly)	All participants for FG and SI
Designing shape to be round with white colour	Pharmacist FG, Doctor 2 SI
Educating children and may be their parents concerning ODTs formulations	Doctor 4 and 5 SI, Nurse 3 SI

FG: Focus group; SI: Semi-structured interview.

Overall, in phase 1 and 2, the majority of respondents (90%) recommended that taste and disintegration time were the most important properties respectively in order to develop and design ODTs formulations. Similarly, various studies stated that taste was an important factor in influencing medication adherence and acceptability in paediatric population [20, 21].

### Phase 3- Online survey

A total of 41 online surveys (study cohort n = 110, response rate 37.3%) were completed. The final online survey consisted of four sections. These were: demographic data including details of different healthcare professionals and participant years’ experience of working with paediatric patient; healthcare professionals views and their preferences for various oral dosage forms (liquids, tablets, capsules and ODTs); HCPs recommendations concerning colour, shape, size, thickness, taste, flavour and disintegration time of tablets; participant feedback about the survey and further recommendations.

### Section one: Demographic results of 41 healthcare professionals

The first section of results displayed the number of respondents from each Hospital. Participants were from multidisciplinary professions. It gives the breakdown of their professions and years’ experience working in paediatrics. Pharmacist were the highest percentage of participants in this phase (46%) followed by nurse (29%) and medical practitioners (24%) respectively. More than half (54%) of the respondents reported their experience ranged from 1 to 5 years. The results showed a significant difference among the different healthcare professionals years of experience (p<0.05) (Table 5).

**Table 5:** Details of healthcare professional’s respondents at Hospital 1 and Hospital 2, including number and percentage of each profession and years of experience with statistical significance also detailed in the final column.

Characteristics		Hospital 2 N = 21 (%)	Hospital 1 N = 20 (%)	Total N = 41 (%)	P value
**Professions**	Consultant (Medical staff)	0 (0.0%)	0(0.0%)	0(0.0%)	<0.05
	Junior medical staff	7(33.3%)	3(15.0%)	10(24.4%)	
	Nurse	3 (14.3%)	9(45.0%)	12(29.3%)	
	Pharmacist	11 (52.40%)	8(40.00%)	19(46.3%)	
**Years of experience**	1–5	10 (47.6%)	12(60.0%)	22(53.7%)	<0.05
	6–10	7 (33.3%)	4(20.0%)	11(26.8%)	
	11–15	2 (9.5%)	0(0.0%)	2(4.9%)	
	16–20	0(0.0%)	3(15.0%)	3(7.3%)	
	>20	2(9.5%)	1(5.0%)	3(7.3%)	

### Section two: Healthcare professionals views regarding paediatric dosage forms

**Fig 2:**
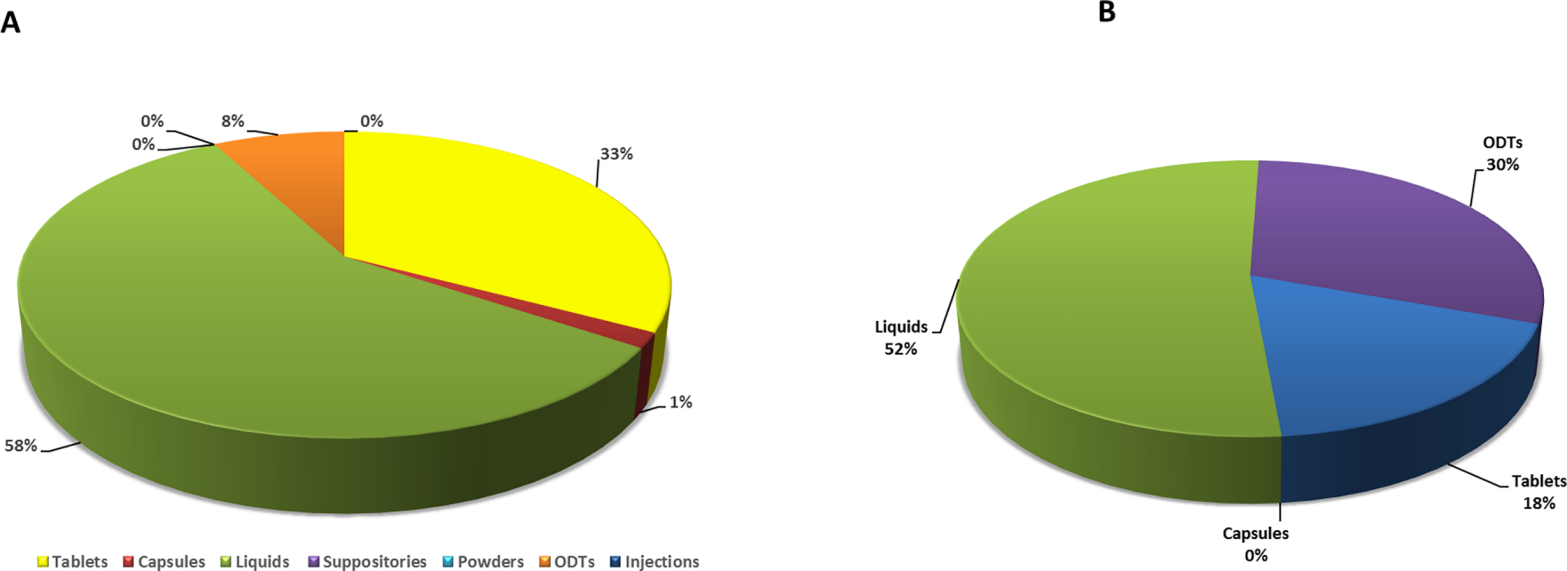
(A) Distribution of the types of dosage forms which are prescribed, supplied or given by healthcare professionals to paediatric patients indicating a massive preference for liquid oral dosage forms, then followed by tablets and (B) Distribution of the preferred oral dosage forms among healthcare professionals, indicating a preference for liquid dosage forms, which is then followed by ODTs and traditional tablets.

This section involves the analysis of the responses of the participants according to types of dosage forms that have been prescribed, supplied or given to paediatric patients. Fig 2A illustrated that liquids dosage forms were thought to be more popular (58%) compared to tablets (33%), ODTs (8%), capsules (1%) and other dosage forms. There was a significant difference among distribution of different dosage forms to children (p<0.05). In this study liquids were the most prescribed dosage forms among dual sites, possibly due to availability as well as shortages for other dosage forms. This was supported by evidence from [22] who indicated that when healthcare professionals were asked to rank the factors that impact their selection of paediatric medicines, availability was the most important factor when prescribing oral medications to children.

**Fig 3:**
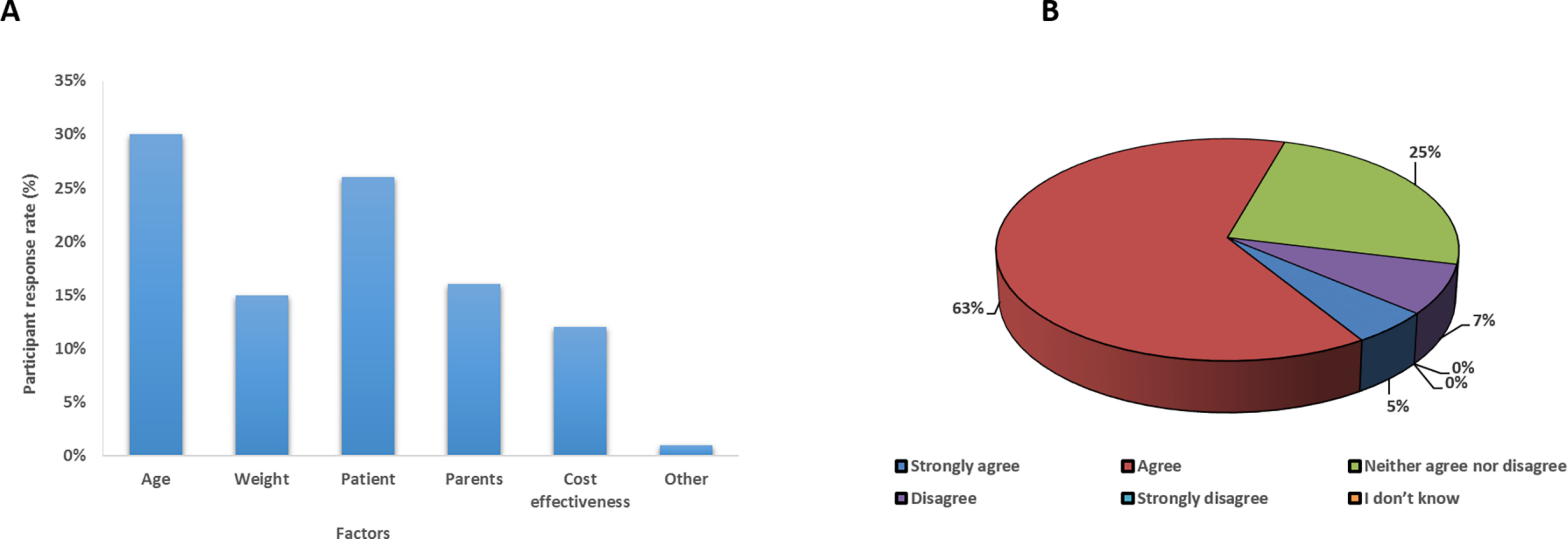
(A) Distribution of factors which influence choice of formulations for paediatric patients indicating that patient age is the most influential factor that dictates the preference of dosage form and (B) Distribution of total respondents’ opinions regarding liquid formulations substitution with ODTs in paediatric patients, indicating that the participants would strongly agree that liquid dosage forms could potentially be replaced with ODTs.

In the previous study, respondents were asked to rank their opinion on the most preferred oral dosage forms, results showed that liquids were the most popular oral dosage form (52%) followed by ODTs (30%), tablets (18%) with no preference for capsules (0%) (Fig 2B). The rational for healthcare professionals on preference of liquids in the pediatric population was possibly due to a number of factors (Fig 3A), including child age and weight, parents, cost effectivness and medicine manipulation. Furthermore, there is a regularly thought bias amongst healthcare professionals that liquids are preferred by younger children [23].

### Section three: ODTs formulations

#### Healthcare experience regarding ODTs

It is essential to mention that ODTs have become a popular area of research for scientists in the last decade as a new ‘drug delivery system’, with benefits including ODTs being a more acceptable dosage form specifically for paediatric patients due to ease of use and administration [24]. Orally disintegrating dosage forms have great promise for paediatric patients as they are easy to administer and don’t require water, with a reduction in choking risk due to the rapid disintegration [1]. Furthermore, previous studies and surveys stated that ODTs are well received by paediatric patients and healthcare professionals [25, 26]. The results in this study (Table 6) showed that a total of 32 (78%) healthcare professionals prescribed/dispensed or administered ODT formulations to children whereas 22% of participants did not prescribe ODTs. Additionally, respondents were asked regarding the number of ODT dosage forms that had been given to patients; approximately half of the respondents (53.7%) followed by only (2.4%) indicated that between 1 to 5 and more than 10 formulations were given over the last 12 months respectively.

**Table 6:** Details of respondents regarding ODTs prescribing/ dispensing or administering and total number of prescribed ODTs over last 12 months.

Have you ever prescribed (doctor)/dispensed and supplied (Pharmacist)/ given or administered (nurse) ODTs to paediatric patients?	In the last 12 months, do you know how many ODTs formulations have you prescribed, dispensed or administered?					Totals
	None	1–5 Formulations	6–10 Formulations	More than 10 Formulations	I don't know	
**Yes**	0.0%	22(53.7%)	5(12.2%)	1(2.4%)	4(9.8%)	32(78.1%)
**No**	8(19.5%)	0(0.0%)	0(0.0%)	0(0.0%)	1(2.4%)	9(22%)
**Not applicable**	0(0.0%)	0(0.0%)	0(0.0%)	0(0.0%)	0(0.0%)	0(0.0%)
**I don't know**	0(0.0%)	0(0.0%)	0(0.0%)	0(0.0%)	0(0.0%)	0(0.0%)
**Totals**	8(19.5%)	22(53.7%)	5(12.2%)	1(2.4%)	5(12.2%)	41(100%)

Interestingly, when healthcare professionals were asked concerning what extent they agree or disagree that liquid formulations could be substituted with ODTs in paediatric patients, approximately (63%) of respondents agreed that a suitable alternative to liquids was the ODT dosage form as shown in (Fig 3B). Similarly, [27] identified that approximately 80% of prescribed liquid formulations could be substituted with a solid dosage forms in children.

Considering ODTs could be an alterative to liquid formulations, healthcare professionals were asked to give their opinions regarding multiple factors such as safety, efficacy, cost effectiveness and compliance. Pharmacists indicated more benefits regarding safety and suggested that “liquid medications may be more likely to have unsuitable excipients for children particularly if they have been formulated for the adult population”. Furthermore, regarding dosing error, liquid formulations require calculation and measurement of the dose volume whereas ODT's are used because they are available in the appropriate dose and don't need further manipulation. They also mentioned that possible risk of accidental overdose for the patient was higher with liquids, for instance, if a young sibling accessed a liquid medicine they may be more likely to consume more than if they accessed ODTs. On the contrary, one pharmacist stated that a lot of solid forms come in very poor dosing variances so the tendency is to dissolve in liquid and give a proportion, but an accurate dose cannot be guaranteed. Lastly, there was a number of issues with children who have feeding tubes and the dispersed tablets blocking them. When asked regarding efficacy, the majority of participants indicated that they had no idea as there were too few ODT formulations available compared to liquids. The vast majority of respondents mentioned that in general, oral solid forms are much less costly than liquid formulations, since they are easier to develop, manufacture, transport, store and distribute. These findings were in line with previous results by [27] who stated that solid dosage forms were more convenient and less costly compared with liquid medications for paediatric patients. With regard to compliance and adherence, respondents recommended that ODT formulations may increase compliance but it depended on the taste. Similarly, a study concluded that tablets for children may be considered as a viable alternative to improve adherence and therefore overall acceptability [28]. Furthermore, research by [29] confirmed that adherence to tablet formulations was significantly superior than liquid formulations.

#### Characteristics of ODTs

**Fig 4:**
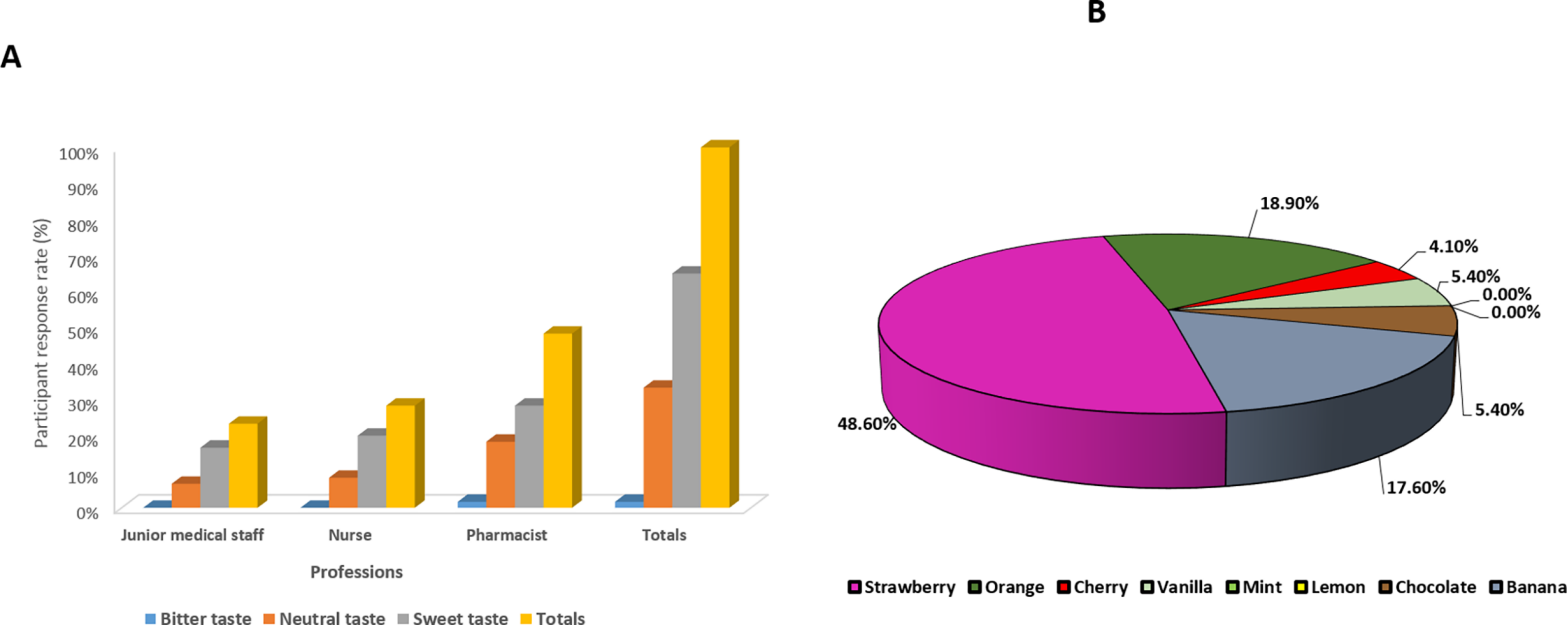
(A) Distribution of preferred ODTs tastes stratified by different professions indicating that sweet taste is preferred by all three sets of healthcare professionals, with neutral taste also being popular and (B) Distribution of preferred ODTs flavours by all respondents, with strawberry flavour being the most popular, followed by orange and banana.

Acceptability and adherence of medication in paediatric patients is potentially affected by characteristics (i.e. taste, flavour, size, shape and colour) of dosage forms [20, 30]. Consequently, the next set of investigations were focused on assessing the healthcare professionals views and their preferences on different attributes of ODTs such as colour, taste, shape, flavour and disintegration time. Healthcare professionals were asked how medicines for children should taste. Their responses–overall and stratified by healthcare profession types are shown in (Fig 4A). The majority of participants (65%) preferred sweet tasting medicines for children followed by neutral/no taste and bitter taste (33% and 2% respectively). There was a statistically significant difference (p<0.05) between acceptable taste by healthcare care professionals. This is in line with findings from Adams [22] who stated that the majority of participants preferred sweet tasting medicines for paediatric populations.

The addition of flavours into formulations not only masks the taste of active ingredients but also improves medication adherence, for instance flavouring medicine increases patient adherence to over 90%, from an average of 50% [23]. In the present study nearly the half of the participants (48.6%) preferred strawberry followed by orange and banana (18.9% and 17.6% respectively) while no preference (0%) was recorded for mint and lemon (Fig 4B). Overall strawberry was the most preferred flavour, significant differences were found among respondent preferences (p<0.05). Similarly, the previous study which was carried out with the paediatric population, confirmed that the participants preferred sweet taste (76.9%) along with strawberry flavour (30.8%) [9].

**Fig 5:**
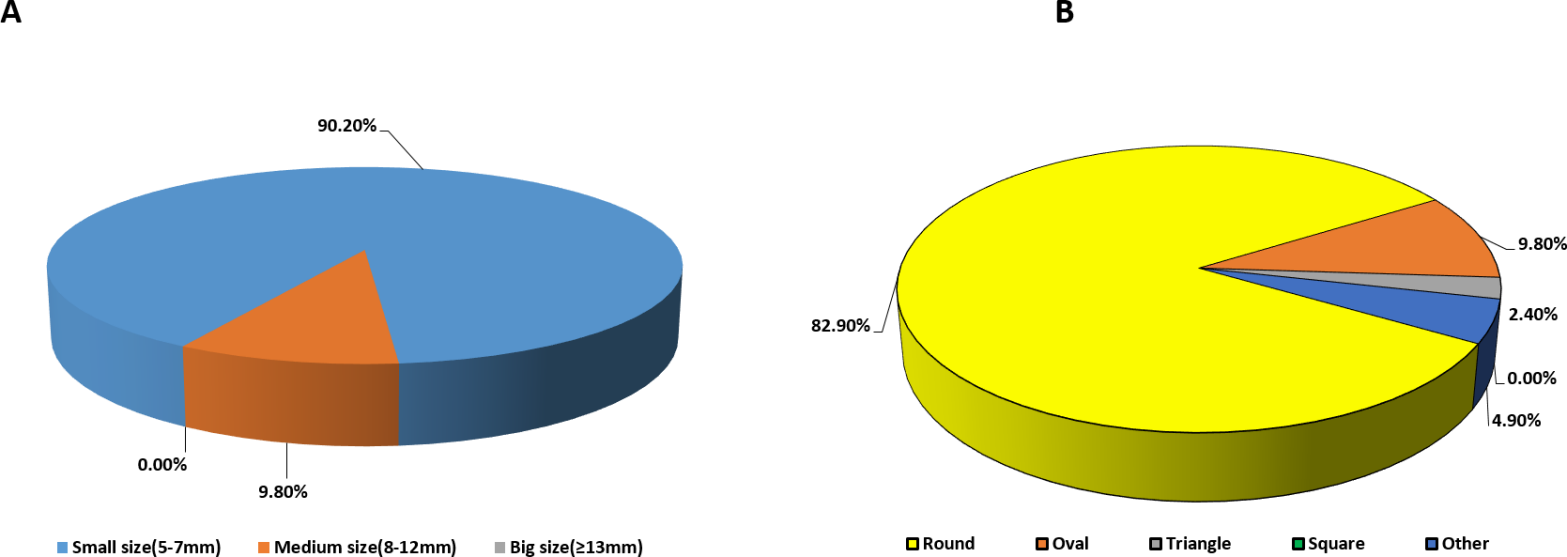
(A) Distribution of preferred ODTs sizes by all respondents clearly showing that a small dosage form would be preferable and (B) Distribution of preferred ODTs shapes by all respondents indicating that a round shape is massively preferred compared to any other tablet shape.

Due to consideration of size and shape of dosage forms, they may affect the transit of the product through the pharynx and oesophagus and may directly affect a patient’s capability to swallow a particular drug product [31]. The current study reported that vast majority (90.2%) of respondents’ preferred small size (5 to 7 mm) compared to the medium 9.8% (8 to 12 mm) or big with 0% preference (≥13mm), as shown in (Fig 5A). The study for opinions in paediatric patients was also in agreement with the opinion of healthcare professionals, as 64.4% of patients preferred small tablets, with large tablets being the least favour (1.9%) [9]. The size of ODTs was potentially highlighted across all groups of healthcare professionals with small size highly recommended. Several studies support the findings and have stated the negative attitudes of children concerning big sizes of tablets [19, 32, 33].

The chart below (Fig 5B) shows the respondents preferences based on the shape of the ODT formulations. The majority of the participants preferred ODTs that are round in shape. This accounted for 82.9% of the responses while the oval shape was second with a preference of 9.8%, approximately 4.9% reported that they had no preference for shape with the least preferred shapes being triangle and square at 2.4% and 0% respectively. Patient preference, as highlighted in the previous study, also identified round shape as the most preferred (35.6%), however patients also indicated the preference to a more profound shape such as a heart (22.1%), with square shaped tablets being negatively perceived (4.7%) [9].

**Fig 6:**
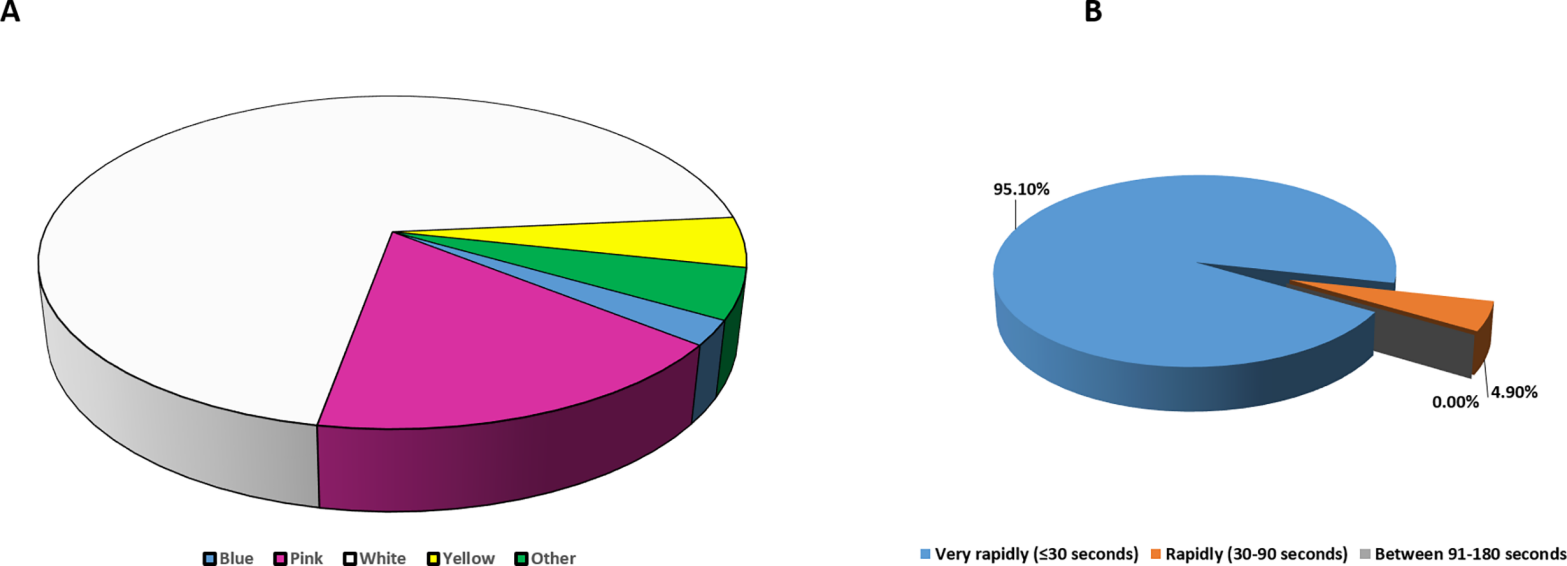
(A) Distribution of preferred ODTs colours by all respondents showing that a white tablet is the most preferred colour amongst the healthcare professionals for paediatric administration and (B) Distribution of preferred ODTs disintegration times by all respondents showing that a rapidly disintegrating tablet would be seen as the ideal ODT by the healthcare professionals.

With respect to colour preferences (Fig 6A) demonstrated that white was the most preferred colour for ODTs by more than 70.0% of the respondents followed by pink (17%), yellow (5%) and finally blue (2%). A significant difference was identified by Chi-square test for colour preferences p<0.05. This was in contrast to the study conducted with paediatric patients, where pink was the preferred colour by 30.8% of patients, which was followed by white (26%) [9]. It was worth mentioning that the selection of an appropriate colouring agent may positively impact child acceptance and also enhance medication adherence [34]. The results in (Fig 6B) showed that the healthcare professionals opinions regarding length of time for ODT formulations to be disintegrated in the mouth, with the vast majority of the participants (95.1%) preferring very rapidly (<30sec) disintegrating ODTs followed by rapidly disintegrating ODTs (between 30 to 90 sec) at 4.9%. The opinions of the healthcare professionals in this study reflected the paediatric population from the previous study [9].

**Fig 7:**
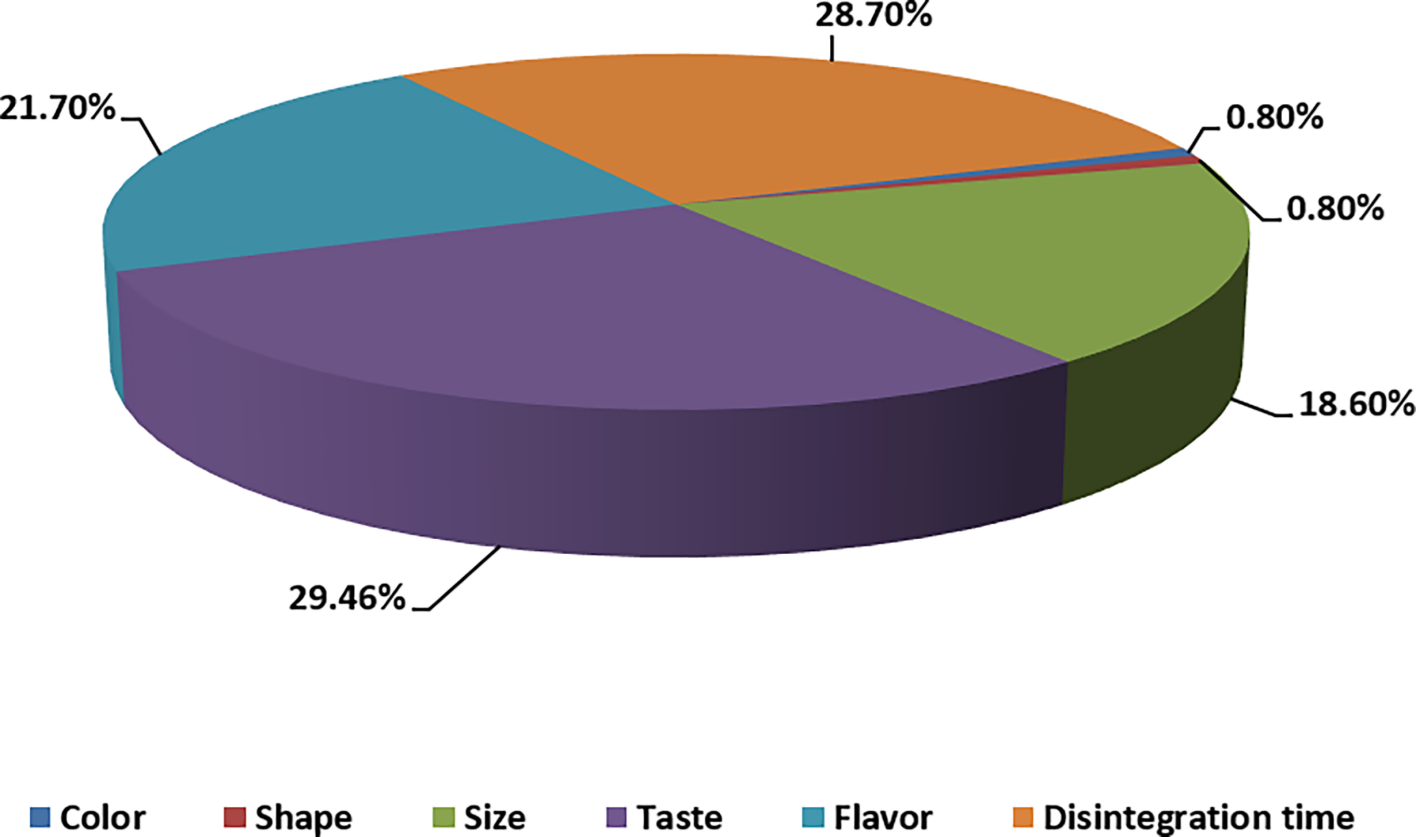
Percentages distribution of the most important characteristics of ODTs. Taste disintegration time and flavour appeared as the most important factors by the healthcare professionals when considering ODTs.

Respondents were also asked what the most important characteristics of ODTs were and the results showed that taste was the most important property (29.5%), followed by disintegration time, flavour and size (28.7%, 21.7% and 18.6% respectively) whereas, colour and shape were the least important characteristics (Fig 7). A significant difference was found between those characters (p<0.05). The results indicated that the most important factor was taste with the findings aligning with the published literature [20, 21, 35]. These results reflected the previous study, which identified disintegration time and taste as the two most important factors according to patient opinion, with flavour and size also being important [9]

### Section four: Further recommendations, feedback and limitations

In the last section of the study, the healthcare professionals were asked to give their opinions, recommendations and feedback on how the study was conducted. The vast majority of respondents designated that regardless of the fact that it was a good idea, there were areas that could be improved. For instance, they pointed out that most questions should be asked to paediatric patients, however this had been covered in the previous study [9]. In addition, pharmacists also indicated that there were very few ODT formulations available for example, Calpol Fast Melts, Maxalt-MLT (Rizatriptan), Nurofen Meltlets and Reglan ODT (Metoclopramide) to enable them to make an informed answer to most of the survey questions [6]. With regard to drug manufacture and design, a few participants recommended that film formulations may be another form of oral dispersible formulations that could offer further advantages. Participants also suggested that taste was the most important property reported for ODT administration, hence the manufacturers should ensure that the taste is neutral to sweet, but not bitter. The recruitment process in focus groups (phase 1) was carried out through pharmacists and nurses but not the medical practitioner group. This may have led to under representation of doctors’ perspectives and input. Certainly some healthcare professionals participating in focus group from the same institution were known to each other, this might have been seen as a potential limitation as respondents may have been more disposed to speak in a ‘socially accepted’ style (i.e. less fairly) [36]. The study was conducted at two sites in the UK, thus it cannot be generalised and viewed as a nationwide perspective, and further exploration in another countries is required.

## Conclusion

In summary, this study identified a plethora of recommendations and opinions for paediatric dosage forms, particularly how ODTs are perceived by healthcare professionals. Secondly, the study identified that HCP perceived suitable organoleptic properties of ODTs (e.g. disintegration time) which influenced acceptability in paediatric patients. As a result this pragmatic study explored healthcare professional’s views and recommendations of the acceptability and characteristic properties of ODT using a mixed methods approach (focus group, semi-structured- interviews and online survey). The overall results from dual sites demonstrated that 58.0% of respondents preferred to prescribe/administer liquid dosage forms form followed by ODTs. Factors found to significantly influence choice of formulations for paediatric patients were age, weight, parent/care giver and cost effectiveness of dosage forms. Although, 63.0% of respondents agreed that liquid formulations could be substituted with ODTs in paediatric patients, the number of available ODTs were insufficient to be prescribed or administered. From the characteristics results in this study, it was concluded that taste, disintegration time and flavour were the most important properties related to ODT administration as highlighted by 29.5%, 28.7% and 21.7% of respondents. Additionally, the other important characteristics of solid dosage forms were white colour, small size, round shape, strawberry flavour and rapid disintegration time. Further studies exploring the opinions of parents concerning paediatric dosage forms would complement this research. This study also suggests that there is a need for further research to develop a wider range of ODTs for use in the paediatric population.

## References

[1] EMEA. Reflection paper: formulations of choice for the paediatric population. EMEA, London 2006; Available from: http://www.ema.europa.eu/docs/en_GB/document_library/Scientific_guideline/2009/09/WC500003782.pdf

[2] Michaud P, Suris J, Viner R. The adolescent with a chronic condition. Part II: healthcare provision. Arch Dis Child, 2004;89(10): 943–949 doi: 10.1136/adc.2003.045377 15383439 10.1136/adc.2003.045377 PMC1719690

[3] Bauman L, Drotar D. A patient-centered approach to adherence: Risks for nonadherence In: Drotar D, editor. Promoting adherence to medical treatment in chronic childhood illness: Concepts, methods, and interventions. Psychology Press; 2000 pp. 71–93.

[4] Patel A, Jacobsen L, Jhaveri R, Bradforf K. Effectiveness of pediatric pill swallowing interventions: a systematic review. Pediatr, 2015;135(5):883–9 10.1542/peds.2014-2114 25896843

[5] Barr H, Helme M, Davray L. Review of Interprofessional Education in the United Kingdom 1997–2013. Centre for the Advancement of Interprofessional Education; 2014.

[6] Hirani J, Rathod A, Vadalia R. Orally disintegrating tablets: a review. Tropical journal of pharmaceutical research, 2009;8(2):161–72

[7] Sumiya K, Baba Y, Inomata S, Toyooka H, Kohda Y. Preparation and clinical evaluation of orally-disintegrating clonidine hydrochloride tablets for paediatric preanesthetic medication. Yakugaku zasshi: Journal of the Pharmaceutical Society of Japan, 2000;120(7):652–6 10920719 10.1248/yakushi1947.120.7_652

[8] Benkert O, Szegedi A, Philipp M, Kohnen R, Heinrich C, Heukels A, et al Mirtazapine orally disintegrating tablets versus venlafaxine extended release: a double-blind, randomized multicenter trial comparing the onset of antidepressant response in patients with major depressive disorder. J Clin Psychopharmacol, 2006;26(1):75–8. 16415711 10.1097/01.jcp.0000194622.99986.d6

[9] Alyami H, Dahmash E, Alyami F, Dahmash D, Huynh C, Terry D, et al Dosage form preference consultation study in children and young adults: paving the way for patient-centred and patient-informed dosage form development. European Journal of Hospital Pharmacy, 2016;24(6):332–7. 10.1136/ejhpharm-2016-001023 PMC6451603 31156967

[10] Mukattash T, Hawwa A, Trew K, Mcelany J. Healthcare professional experiences and attitudes on unlicensed/off-label paediatric prescribing and paediatric clinical trials. Eur J Clin Pharmacol, 2011;67(5):449–61. doi: 10.1007/s00228-010-0978-z 21243345 10.1007/s00228-010-0978-z

[11] Akram G, Mullen A. Paediatric nurses' knowledge and practice of mixing medication into foodstuff. Int J Pharm Pract, 2012;20(3):191–8. doi: 10.1111/j.2042-7174.2011.00179.x 22554162 10.1111/j.2042-7174.2011.00179.x

[12] Gale N, Heath G, Cameron E, Rashid S, Redwood S. Using the framework method for the analysis of qualitative data in multi-disciplinary health research. BMC Med Res Methodol, 2013 13(1): p. 117. 24047204 10.1186/1471-2288-13-117 PMC3848812

[13] Walsh D, Downe S. Meta‐synthesis method for qualitative research: a literature review. J Adv Nurs, 2005;50(2):204–211. doi: 10.1111/j.1365-2648.2005.03380.x 15788085 10.1111/j.1365-2648.2005.03380.x

[14] Ritchie J, Spencer L. Qualitative data analysis for applied policy research. In Bryman A, Burgess RG, editors. Analysing qualitative data; 1994 pp 173–194

[15] Siccama C, Penna S. Enhancing validity of a qualitative dissertation research study by using NVivo. Qualitative research journal, 2008;8(2):91–103.

[16] Brooks J, Mccluskey S, Turley E, King N. The utility of template analysis in qualitative psychology research. Qual Res Psychol, 2015;12(2):202–222. doi: 10.1080/14780887.2014.955224 27499705 10.1080/14780887.2014.955224 PMC4960514

[17] Ilieva J, Baron S, Healey N. Online surveys in marketing research: Pros and cons. International Journal of Market Research, 2002;44(3):361–376.

[18] Ricci G, Netto E, Luz E, Rodamilans C, Brites C. Adherence to antiretroviral therapy of Brazilian HIV-infected children and their caregivers. Braz J Infect Dis, 2016;20(5):429–436. doi: 10.1016/j.bjid.2016.05.009 27471126 10.1016/j.bjid.2016.05.009 PMC9425490

[19] Roberts K. Barriers to antiretroviral medication adherence in young HIV-infected children. Youth & Society, 2005;37(2):230–245.

[20] Squires L, Lombardi D, Sjostedt P, Thompson C. A systematic literature review on the assessment of palatability and swallowability in the development of oral dosage forms for pediatric patients. Therapeutic Innovation and Regulatory Science, 2013;47(5):533–541. 10.1177/2168479013500288 30235574

[21] Matsui D. Assessing the palatability of medications in children. Paediatric and Perinatal Drug Therapy, 2007;8(2):55–60.

[22] Adams L, Craig S, Mmbag E, Naburi H, Lahey T, Nutt C, et al Children’s medicines in Tanzania: a national survey of administration practices and preferences. PLoS One, 2013;8(3):e58303 doi: 10.1371/journal.pone.0058303 23484012 10.1371/journal.pone.0058303 PMC3590153

[23] Bryson P. Patient-centred, administration friendly medicines for children–An evaluation of children’s preferences and how they impact medication adherence. Int J Pharm, 2014;469(2):257–259. doi: 10.1016/j.ijpharm.2014.04.069 24797763 10.1016/j.ijpharm.2014.04.069

[24] Parkash V, Maan S, Yadav S, Jogpal V. Fast disintegrating tablets: Opportunity in drug delivery system. J Adv Pharm Technol Res, 2011;2(4):223 doi: 10.4103/2231-4040.90877 22247889 10.4103/2231-4040.90877 PMC3255350

[25] MacGregor A, Brandes J, Eikermann A. Migraine prevalence and treatment patterns: the global Migraine and Zolmitriptan Evaluation survey. Headache, 2003;43(1):19–26. 12864754 10.1046/j.1526-4610.2003.03004.x

[26] Carnaby G, Crary M. Pill swallowing by adults with dysphagia. Arch Otolaryngol Head Neck Surg, 2005;131(11):970–5. doi: 10.1001/archotol.131.11.970 16301368 10.1001/archotol.131.11.970

[27] Lajoinie A, Henin E, Kassai B, Terry D. Solid oral forms availability in children: a cost saving investigation. Br J Clin Pharmacol, 2014;78(5):1080–9. doi: 10.1111/bcp.12442 24965935 10.1111/bcp.12442 PMC4243883

[28] Ansah E, Gyapong J, Agyepong I, Evans D. Improving adherence to malaria treatment for children: the use of pre‐packed chloroquine tablets vs. chloroquine syrup. Trop Med Int Health, 2001;6(7):496–504. 11469941 10.1046/j.1365-3156.2001.00740.x

[29] Bagenda A, Mosha L, Bagenda D, Sakwa R, Fowler M, Musoke P. Adherence to tablet and liquid formulations of antiretroviral medication for paediatric HIV treatment at an urban clinic in Uganda. Ann Tropical Paediatr, 2011; 31(3):235–245. 10.1179/1465328111Y.0000000025 21781419

[30] Cheng A, Ratnapalan S. Improving the palatability of activated charcoal in pediatric patients. Pediatr Emerg Care, 2007;23(6):384–386. doi: 10.1097/01.pec.0000278402.45321.f6 17572522 10.1097/01.pec.0000278402.45321.f6

[31] Jacobsen L, Riley K, Lee B, Bradford K, Jhaveri R. Tablet/Capsule Size Variation Among the Most Commonly Prescribed Medications for Children in the USA: Retrospective Review and Firsthand Pharmacy Audit. Pediatr Drugs, 2016;18(1):65–73. 10.1007/s40272-015-0156-y 26801779

[32] Paranthaman K, Kumarasamy N, Bella D, Webster P. Factors influencing adherence to anti-retroviral treatment in children with human immunodeficiency virus in South India–a qualitative study. AIDS care, 2009;21(8):1025–1031. doi: 10.1080/09540120802612857 20024759 10.1080/09540120802612857

[33] Reddington C, Joyce C, Arlene B, Maripat T, Dorothy S, Catherine K, et al Adherence to medication regimens among children with human immunodeficiency virus infection. Pediatr Infect Dis J, 2000;19(12):1148–53. 11144374 10.1097/00006454-200012000-00005

[34] Levitan C, Zampini M, Li R, Spence C. Assessing the role of color cues and people's beliefs about color–flavor associations on the discrimination of the flavor of sugar-coated chocolates. Chem Senses, 2008;33(5):415–423. doi: 10.1093/chemse/bjn008 18310060 10.1093/chemse/bjn008

[35] Hasamnis A, Patil S, Kumar A, Thu K, Mohanty B. Preferences of medical students regarding physical characteristics of oral solid dosage formulations in Malaysia. Journal pharmacol Pharmacother, 2011;2(2):119–120. 10.4103/0976-500X.81909 PMC3127342 21772776

[36] Rabiee F. Focus-group interview and data analysis. Proc Nutr Soc, 2004;63(4):655–660. 15831139 10.1079/pns2004399

